# Exact and parameterized algorithms for choosability

**DOI:** 10.1007/s00236-025-00492-0

**Published:** 2025-05-30

**Authors:** Ivan Bliznets, Jesper Nederlof

**Affiliations:** 1 Faculty of Science and Engineering, University of Groningen, Nijenborgh 9, Groningen, 9747 AG Groningen Netherlands; 2 Department of Information and Computing Sciences, Utrecht University, Princetonplein 5, Utrecht, 3584 CC Utrecht Netherlands

## Abstract

In the Choosability problem (or list chromatic number problem), for a given graph *G*, we need to find the smallest *k* such that *G* admits a list coloring for any list assignment where all lists contain at least *k* colors. The problem is tightly connected with the well-studied Coloring and List  Coloring problems. However, the knowledge of the complexity landscape for the Choosability problem is pretty scarce. Moreover, most of the known results only provide lower bounds for its computational complexity and do not provide ways to cope with the intractability. The main objective of our paper is to construct the first non-trivial exact exponential algorithms for the Choosability problem, and complete the picture with parameterized results. Specifically, we present the first single-exponential algorithm for the decision version of the problem with fixed *k*. This result answers an implicit question from Eppstein on a stackexchange thread discussing upper bounds on the union of lists assigned to vertices. We also present a $2^{n^2} poly(n)$ time algorithm for the general Choosability problem. In the parameterized setting, we give a polynomial kernel for the problem parameterized by vertex cover, and algorithms that run in FPT time when parameterized by a size of a clique-modulator and by the dual parameterization $n-k$. Additionally, we show that Choosability admits a significant running time improvement if it is parameterized by cutwidth in comparison with the parameterization by treewidth studied by Marx and Mitsou [ICALP’16]. On the negative side, we provide a lower bound parameterized by a size of a modulator to split graphs under assumption of the Exponential Time Hypothesis.

## Introduction

For an undirected graph *G*, we call *k* a choice number of *G* (or choosability number) if under any list assignment of length at least *k* to the vertices, it is possible to find a proper list coloring. In the Choosability problem, the goal is to find a choice number of an input graph. Sometimes the choosability number is also called the list chromatic number. Choosability was introduced by Vizing [27] and independently by Erdős, Rubin, and Taylor [9]. It is known that the problem is $\Pi _2^p$-complete even for very restricted classes of graphs. For example, $k$-Choosability (the version of Choosability where *k* is a fixed parameter) is $\Pi _2^p$-complete in bipartite graphs for any *k* [18], 4-Choosability is $\Pi _2^p$-complete in planar graphs [17], 3-Choosability is $\Pi _2^p$-complete in planar triangle-free graphs [17] and in graphs that are a union of two forests [17]. Moreover, $3$-Choosability is one of the first natural problems for which a double exponential lower bound parameterized by treewidth was established [23]. There is an enormous amount of combinatorial results for Choosability [9, 1,2, 6, 22, 24,25]. Generally, these results provide bounds on the choice number or describe situations where a graph is *k*-choosable. However, the number of algorithmic results is surprisingly small. Besides the mentioned lower bounds on the computational complexity of the problem, the following algorithmic results were established: $k$-Choosability is Fixed Parameter Tractable (for short FPT, recall this means that instances (*x*, *k*) of the problem can be solved $f(k)|x|^{O(1)}$ time for some function *f*) in $P_5$-free graphs parameterized by *k* [15, 16], $k$-Choosability admits a linear algorithm for fixed *k* on *H*-free graphs where *H* is a disjoint union of paths [14], Choosability admits an FPT-algorithm parameterized by treewidth [23, 11].

One might expect that the Choosability problem has a similar behavior to Coloring or List  Coloring problems, and that results for these problems can be easily carried over to Choosability. Surprisingly, this is not the case. For example, List  Coloring is *W*[1]-hard parameterized by treewidth (even parameterized by vertex cover) [11, 7], while Choosability admits an FPT-algorithm [23, 11] (we recall that it is unlikely that *W*[1]-hard problems admit FPT-algorithms). The addition of a universal vertex always increases the chromatic number but this is not true for the choice number. Moreover, even the choice number of bipartite graphs is unbounded, see Lemma 1. *k*-Coloring and List  Coloring with lists’ length bounded by *k* are trivially solvable in $\mathcal {O}^*(k^n)$ time. However, it is not trivial to construct a single-exponential algorithm for $k$-Choosability for a fixed *k*. The existence of a single-exponential algorithm for $k$-Choosability implicitly was asked by Eppstein in a stackexchange post.^1^ There, it was noted that it is easy to construct such an algorithm if we know that the total number of different colors used in lists is bounded by some function *f*(*k*). Unfortunately, it was shown that there are no such functions [22], and our algorithm cannot build on this argument. Nevertheless, we show:

### Theorem 1

Let *G* be an undirected graph on *n* vertices and *k* be an integer given as input.

1. $k$-Choosability can be solved in $f(k)^{O(n)}$ for some function *f*,
2. Choosability can be solved in $\mathcal {O}^*({2^{n^2}})$ time.

The constructed single-exponential algorithm is based on the fact that in *k*-choosable graphs the average degree is bounded by some function *f*(*k*). Our second exact exponential algorithm for the the choice number with running time $2^{n^2} poly(n)$ provides an improvement upon a slightly naive $2^{\mathcal {O}(n^2 \log n)}$ time algorithm. It should be noted that this run time does not show up often for graph problems; one exception we are aware of is the List Edge Coloring problem [21]. A key combinatorial ingredient of our algorithm is the fact that in some sense we can bound the total number of colors by *n*. We note that the obtained running time is significantly worse than the running time of the Subset Convolution algorithm for the Coloring and List  Coloring problems from [3]. That is why one can consider the question whether there exists an improvement on the $2^{n^2} poly(n)$ time algorithm or a lower bound that excludes such improvement based on the Exponential Time Hypothesis (ETH) to be an interesting open question.

In this paper, we also consider different parameterizations of Choosability. The most important such result is for Choosability parameterized by cutwidth. Specifically, we prove the following theorem.

### Theorem 2

Let *G* be an undirected graph on *n* vertices given along with a cutwidth ordering of width *w*. Then Choosability can be solved in time $2^{2^{O(w)}}n$ time.

We note that parameterization by cutwidth allows us to obtain a significant improvement over the parameterization by treewidth for which only a $2^{k^{O(tw)}} poly(n)$ time algorithm is known [23, 11]. We know that for parameterization by cutwidth, there is a matching lower bound under the assumption of the ETH (however, the result is not published yet [5]). To prove Theorem 2, we build on the method from [20] that computes the chromatic number of a graph in $O(2^w n)$ time, when given a cutwidth ordering of width *w*. That algorithm used an argument based on matrix rank to show the table size of the natural dynamic programming algorithm can be sparsified (i.e. we view a dynamic programming table as a family of partial solutions, and with sparsifying we mean that some partial solutions are omitted) in such a way that global solutions remain detected. We show that this approach can also be employed for the Choosability problem.

We also provide a polynomial *kernel* for $k$-Choosability parameterized by vertex cover if *k* is fixed. Recall a *g*(*p*)-kernel is a polynomial time algorithm that, given an instance *x* with parameter *p*, outputs an equivalent instance of the same problem of size (measured here in terms of the number of vertices) *g*(*p*). Formally we prove the following.

### Theorem 3

$k$-Choosability admits a kernel with $O(w^k)$ vertices for a fixed integer *k*, where *w* is the size of a vertex cover of the input graph *G*.

Using Ohba’s Conjecture (proved by Noel et al. in [25]) that connects the chromatic number with the choice number in case of a very large chromatic number, we construct an FPT-algorithm for Choosability parameterized by a size of a *clique-modulator* (Choosability is trivial on complete graphs as $ch(K_n)=n$) and a *linear compression* for $k$-Choosability parameterized by $n-k$ (*n*-Choosability is trivial as clearly $ch(G)\le n$). Here the term $\mathcal {G}$-modulator of a graph *G*, for some graph class $\mathcal {G}$, refers to a vertex subset *X* of *G* such that $G-X$ is in $\mathcal {G}$. A *g*(*p*)-compression is a polynomial time algorithm that, given an instance *x* with parameter *p*, outputs an equivalent instance of a possibly different computational problem of size (measured here in terms of the number of vertices) *g*(*p*).

Precisely, we prove the following FPT algorithms that show some instances of Choosability*can* be solved efficiently:

### Theorem 4

$k$-Choosability under the dual parametrization by $n-k$ can be compressed into an instance of $k$-Choosability or into an instance of Dual-Coloring. Moreover, number of vertices in the compressed instance is at most $3(n-k)$.

### Theorem 5

Choosability admits an FPT-algorithm parameterized by the size of clique-modulator *d*.

Given Theorem 5 and the easy observation that Choosability is solvable in polynomial time on split graphs (recall, split graphs are graphs whose vertex set can be partitioned in one clique and one independent set), one may wonder whether Choosability also admits an FPT-algorithm. While we do not rule out this possibility, we present evidence suggesting that the existence of a single exponential parameterized algorithm is unlikely in this case.

We employ Ohba’s Conjecture to show a lower bound for Choosability assuming ETH, to temper expectation here:

### Theorem 6

Unless ETH fails there is no constant $\theta$ such that Choosability is solvable in $\mathcal {O}^*(\theta ^d)$ time where $\text {d}$ denotes size of a modulator to split graphs.

Taking into account the tractability of Choosability on graphs of bounded treewidth, it is natural to study this problem using parameters that can be significantly smaller than treewidth. One of these parameters is cliquewidth. We prove the following result:

### Theorem 7

Choosability does not admit an FPT algortihm parameterized by cliquewidth unless $FPT=W[1]$.

*Organization of the paper*

The rest of this paper is organized as follows. In Sect. 2, we set up the notation. In Sect. 3, we present exact exponential algorithms for $k$-Choosability and Choosability (Theorem 1). Sect. 4 is dedicated to $k$-Choosability parameterized by cutwidth (Theorem 2). In Sect. 5, we provide a linear compression for $k$-Choosability parameterized by $n-k$ (Theorem 4), an FPT-algorithm for Choosability parameterized by a size of a clique-modulator (Theorem 5), and lower bounds for Choosability parameterized by a size of modulator to split graphs (Theorem 6) and cliquewidth (Theorem 7).

## Preliminaries

In this paper, we consider only simple graphs. Used notation for graphs is standard and can be found in [8]. We also use standard definitions (FPT-algorithm, kernel, compression) and notation from parameterized complexity, for details we refer the interested reader to the textbook [7]. For a natural number *k*, we denote by [*k*] the set $\{1, \dots, k\}$. Generally by *n* we denote number of vertices in a graph unless stated otherwise and if the graph is clear from the context. Similarly, by *m* we denote number of edges in a graph. $N_G(v)$ denotes the open neighborhood of vertex *v* in a graph G, we omit subscript *G* if the graph is clear from the context.

For a graph *G*, function $c: V(G) \rightarrow C$ for some set *C* is called a proper coloring if for any edge $uw\in E(G)$ we have $c(u) \ne c(w)$. If a proper coloring exists for the set $C=[k]$, then we call graph *k*-colorable. For a graph *G*, we call a function $\mathcal {L}$ a *list assignment* if for each vertex $v\in V(G)$ the function assigns a set/list of colors. For a list assignment $\mathcal {L}$, we call graph $\mathcal {L}$-colorable if there is a proper coloring *c* of vertices such that for any vertex *v* we have $c(v) \in \mathcal {L}(v)$. A graph *G* is called *k-choosable* if it is $\mathcal {L}$-colorable for any list assignment $\mathcal {L}$ such that $|\mathcal {L}(v)|=k$ for any $v\in V(G)$. *The choosability number (choice number, list chromatic number)* of a graph *G* is the smallest integer *k* such that *G* is *k*-choosable. By *ch*(*G*) we denote the choosability number of the graph *G*. Similarly, by $\chi (G)$ we denote chromatic number of the graph *G*, i.e. the smallest integer *k* such that *G* is *k*-colorable. As one of the central studied parameters is cutwidth we provide its definition below.

### Definition 1

The cutwidth of a graph *G* is the smallest *k* such that its vertices can be arranged in a sequence $v_1, \dots, v_n$ such that for every $i \in [n-1]$, there are at most *k* edges between $\{v_1, \dots, v_i\}$ and $\{v_{i+1}, \dots, v_n\}$.

A *split graph* is a graph *G* such that $V(G)=C \cup I$, $C \cap I =\emptyset$, *G*[*C*] is a clique, *G*[*I*] is an independent set. We call a subset of vertices *D* in a graph *Ga modulator to split graphs* if subgraph $G\setminus D$ is a split graph.

Formal definitions of Dual-Coloring, List  Coloring, $k$-Choosability, Choosability can be found below.

**Table:** 

Dual-Coloring	
**Input**: graph G and an integer k.	
**Question**: Is graph G $(n-k)$-colorable?

**Table:** 

List Coloring	
**Input**: A graph G and a list assignment $\mathcal {L}$.	
**Question**: Is graph G $\mathcal {L}$-colorable?

**Table:** 

k-Choosability	
**Input**: A graph G.	
**Question**: Is graph G k-choosable?

**Table:** 

Choosability	
**Input**: A graph G.	
**Question**: What is the smallest k such that G is k-choosable?	

For the construction of a polynomial kernel, we are using the fact that bipartite graph $K_{k,k^k}$ is not *k*-choosable. It is a well known fact, but nevertheless we present a proof in order to provide some intuition about the Choosability problem.

### Lemma 1

The bipartite graph $K_{k,k^k}$ is not *k*-choosable.

### Proof

Let $A = \{v_1,v_2,\dots, v_k\}$ be the left part and *B* be the right part containing $k^k$ vertices. Consider the following list assignment: vertex $v_i$ for $i\in [k]$ has a list $c_{(i-1)k+1}, c_{(i-1)k+2}, \dots, c_{(i-1)k+k}.$ We assume that $c_i\ne c_j$ for all $i\ne j$. Each vertex from the set *B* has a unique list which contains exactly one color from each list ${{\,\mathrm{\mathcal {L}}\,}}(v_1),{{\,\mathrm{\mathcal {L}}\,}}(v_2),\dots, {{\,\mathrm{\mathcal {L}}\,}}(v_k)$. It is easy to see that no matter how we color part *A* there will be a vertex in part *B* that cannot be colored, since the number of possible lists with exactly one element from each list ${{\,\mathrm{\mathcal {L}}\,}}(v_1),{{\,\mathrm{\mathcal {L}}\,}}(v_2),\dots, {{\,\mathrm{\mathcal {L}}\,}}(v_k)$ is $k^k$. So under this list assignment $K_{k,k^k}$ does not admit proper list-coloring. Hence, $K_{k,k^k}$ is not *k*-choosable. $\square$

Lemma 1 shows that choice number and chromatic number can be very different. However, in some situations values of these numbers coincide. In these cases we can determine the choice number by finding the chromatic number. One of such cases is described by Ohba’s conjecture, proved by Noel, Reed, Wu [25].

### Theorem 8

If $|V(G)| \le 2\chi (G) + 1$, then *G* is chromatic-choosable, i.e. $ch(G)=\chi (G)$.

## Exact algorithms

In this section we provide exact exponential algorithms for $k$-Choosability and Choosability. Before we proceed we provide some definitions and lemmas mainly used only in this section.

For a list assignment $\mathcal {L}$, we denote by $\mathcal {L}[c]$ the induced subgraph of *G* that contains all vertices *v* such that $c \in \mathcal {L}(v)$.

### Lemma 2

If a graph *G* is not $\mathcal {L}$-colorable for some list assignment $\mathcal {L}$, then there is a list assignment $\mathcal {L'}$ such that:

1. $$|\mathcal {L'}(v)|=|\mathcal {L}(v)|$$ for all $$v \in V(G),$$
2. for any color *c* the induced subgraph $\mathcal {L'}[c]$ is a connected subgraph in *G*,
3. *G* is not $\mathcal {L'}$-colorable.

### Proof

If for some *c* the subgraph $\mathcal {L}[c]$ contains $p>1$ connected components $C_1, C_2, \dots, C_p$, then we create new colors $c^1, \dots, c^p$, and for $v \in C_i$ in $\mathcal {L'}$ we use color $c^{i}$ instead of color *c* like it is in the assignment $\mathcal {L}$. Clearly, for any color $c'$, the induced subgraph $\mathcal {L'}[c']$ is connected. If the graph *G* admits a list-coloring under assignment $\mathcal {L'}$, then it also admits list-coloring under the list assignment $\mathcal {L}$. Namely, it is enough to re-color vertices colored in $c^i$ into color *c*. Hence, if *G* is not $\mathcal {L}$-colorable then it is also not $\mathcal {L'}$-colorable. $\square$

We need the following theorem bounding the average degree in a *k*-choosable graph [1].

### Theorem 9

([1]) Let *G* be a simple graph with average degree at least *d*. If $$d> 4 \left( {\begin{array}{c}k^4\\ k\end{array}}\right) \log (2 \left( {\begin{array}{c}k^4\\ k\end{array}}\right) )$$ and *k* is an integer, then $ch(G)> k$.

Now we have all ingredients to prove main result of this section. We will first restate it for convenience:

### Theorem 1

Let *G* be an undirected graph on *n* vertices and *k* be an integer given as input.

1. $k$-choosability can be solved in $f(k)^{O(n)}$ for some function *f*,
2. choosability can be solved in $\mathcal {O}^*({2^{n^2}})$ time.

### Proof

Throughout the proof we identify colors with integers. We assume that $V(G)=\{v_1, v_2, \dots, v_n\}$.

First of all we present an algorithm for $k$-Choosability with $f(k)^{O(n)}$ running time. Recall that if a graph *G* is not *k*-choosable, then there is a list assignment $\mathcal {L'}$with $|\mathcal {L}'( v_i)| = k, \forall i \in [n]$ such that the graph *G* is not $\mathcal {L'}$-colorable. We show how to find such an assignment in $2^{O(n)}$ running time. By Lemma 2 we can assume that for each color *c* the graph $\mathcal {L'}[c]$ is connected. Without loss of generality we can consider only assignments that satisfy the following property: if $p \in \mathcal {L'}(v_i)$, then for any positive integer $q<p$ there is $j\le i$ such that $q \in \mathcal {L'}(v_j)$ (otherwise we can simply rename colors). Under this assumption, for example, we have that $\mathcal {L}'(v_1)=\{1,2,3, \dots, k\}$. Moreover, $\mathcal {L}'$ with this property satisfy the following: for any *l* we have $\mathcal {L'}(v_1) \cup \mathcal {L'}(v_2) \cup \dots \cup \mathcal {L'}(v_l) =\{1,2,3,\dots, c'\}$ for some positive integer $c'$. For each *i*, we order colors in $\mathcal {L'}(v_i)$ in increasing order. Denote by $\mathcal {L'}(v_i)[\ell ]$ the $\ell$-th element in the list $\mathcal {L'}(v_i)$. Taking into account that list $\mathcal {L'}(v_i)$ is ordered we have $\mathcal {L'}(v_i)[1]< \mathcal {L'}(v_i)[2]< \dots < \mathcal {L'}(v_i)[k]$.

Taking into account Theorem 9 we may assume that our input graph has at most $$4 ({\begin{array}{l}k^4\\ k\end{array}}) \log (2({\begin{array}{l}k^4\\ k\end{array}})) n$$ edges, otherwise *G* is not *k*-choosable. Note that the number $$4 ({\begin{array}{l}k^4\\ k\end{array}}) \log (2({\begin{array}{l}k^4\\ k\end{array}}) )n$$ is $\mathcal {O}(n)$ for a fixed integer *k*.

For a list assignment $\mathcal {L}$ and edge $e=v_iv_j \in E(G)$, we denote by $S^{\mathcal {L}}_{ij}$ a set of pairs $\{(a_1,b_1), (a_2, b_2), \dots, (a_{\ell }, b_{\ell })\}$ such that $\mathcal {L}(v_i)[a_q]=\mathcal {L}(v_j)[b_q]$ for all $q \in [\ell ]$. Note that without loss of generality we can assume that $a_1<a_2<\dots <a_{\ell }$ and $b_1<b_2<\dots <b_{\ell }$. This means that the number of such sets of pairs is bounded by $$\sum\nolimits_{\ell =1}^{k} {({\begin{array}{l}k\\ \ell \end{array}}) }^2\le 2^{2k}$$. In order to determine $\mathcal {L'}$, for each edge $v_iv_j$ we consider all possible values of $S^{\mathcal {L}'}_{ij}$. It creates at most $(2^{2k})^{|E(G)|}$ sub-branches which is $2^{O(n)}$ for a fixed *k*. Knowing the $S^{\mathcal {L}'}_{ij}$ for each edge $v_iv_j$ and the fact that for each $t \in [n]$ we have $\mathcal {L'}(v_1) \cup \mathcal {L'}(v_2) \cup \dots \cup \mathcal {L'}(v_t) =\{1,2,3,\dots, c'\}$ for some positive integer $c'$ we can recover $\mathcal {L'}$. Indeed, having this information we can identify induced subgraphs $\mathcal {L}'[1],\mathcal {L}'[2], \dots, \mathcal {L}'[k]$. After that we can find the smallest *i* such that $k+1\in \mathcal {L}'(v_i)$. Having information on edges about common colors on endpoints we find $\mathcal {L}'[k+1]$, since subgraph $\mathcal {L}'[k+1]$ is connected. Then we repeat the process for color $k+2$ and so on. Finally, we realize that either our branch is not valid and such list assignment do not exists or we obtain a list assignment. We check that the obtained assignment $\mathcal {L'}$ contains exactly *k* colors for each vertex. Recall that list coloring can be solved in $\mathcal {O}^*(2^n)$. It is enough to use a subset convolution method [4] for this. Therefore, after recovering $\mathcal {L'}$ from $S_{ij}$ we run the algorithm for list-coloring and check if *G* is $\mathcal {L'}$-colorable or not. If *G* is not $\mathcal {L'}$-colorable, then *G* is not *k*-choosable. However, if in all our subbranches we either failed to recover $\mathcal {L'}$ or *G* was $\mathcal {L'}$-colorable, then we conclude that *G* is *k*-choosable. So, the overall running time is at most $\mathcal {O^*}((2^{2k})^{|E(G)|}\cdot 2^n)$, which is sufficient since $|E(G)|=O(n)$ for fixed *k*.

Now we provide an algorithm for Choosability with $\mathcal {O}^*(2^{n^2})$ running time. It is known [26] that if a graph *G* is not *k*-choosable for some *k*, then there is a list assignment $\mathcal {L}$ containing at most $n-1$ colors in the union $\mathcal {L}(v_1) \cup \mathcal {L}(v_2) \cup \dots \cup \mathcal {L}(v_n)$ such that for each vertex *v* we have $|\mathcal {L}(v)|=k$ and *G* is not $\mathcal {L}$-colorable. Therefore, in order to find such $\mathcal {L}$ it is enough to brute-force all $2^n$ possibilities of $\mathcal {L}[i]$ for each color $i\in [n-1]$. This step creates $2^{n(n-1)}$ subcases, we eliminate those that have unequal length of lists assigned to vertices and then run the $\mathcal {O}^*(2^n)$ time algorithm for list coloring in each subcase. Overall, the running time will be at most $\mathcal {O}^*(2^{n^2})$. $\square$

## Cutwidth

The goal of this section is to prove the following theorem.

### Theorem 2

Let *G* be an undirected graph on *n* vertices given along with a cutwidth ordering of width *w*. Then Choosability can be solved in time $2^{2^{O(w)}}n$ time.

We want to solve *k*-choosability on graphs of cutwidth *w*. Let *q* be the maximum assigned number showing up in a list. In Lemma 1 from [11] it was shown that it is enough to consider $q\le (2tw+1)k<4kw$ (*tw* is the treewidth of the graph) which is at most 4*kw* since cutwidth is larger than treewidth. We can assume $k \le w$ since otherwise the answer is automatically yes (because a greedy algorithm can always find a list coloring if each list is of size at least $w+1$). Hence, we can assume that *q* is at most $4 w^2$. Let $\{v_1,\ldots,v_n\}=V(G)$ be an ordering of cutwidth at most *w*, and let $G_i$ be the prefix graph $G[\{v_1,\ldots,v_i\}]$. Denote $V_i=\{v_1,\ldots,v_i\}$ for the set of vertices of $G_i$. Consider the *i*’th cut and the corresponding bipartite graph $H_i$ with sides $L_i$ and $R_i$. Thus, $|L_i|,|R_i|\le|E(H_i)| \le w$, and $E(H_i)=\{\{v_h,v_j\}: h \le i < j\}$. Note that $L_i \subseteq L_{i-1} \cup \{v_i\}$ and $R_{i-1} \subseteq R_{i} \cup \{v_i\}$.

We will use the following standard vector and function notation: For sets *A*, *B*, we let $A^B$ denote the set of vectors with values from *A* indexed by *B*. We will interchangeably address these vectors as functions $f: B \rightarrow A$ and use function notation $f(\cdot )$ and its inverse $f^{-1}(\cdot )$ (if it exists). For $f \in A^B$ (or thus, equivalently, $f:B\rightarrow A$), we denote the *projection*$f_{|B'}$ for the unique vector $f' \in A^{B'}$ that agrees with *f* on all its values, i.e. $f'(b)=f(b)$ for all $b \in B'$. If $f:B_1 \rightarrow A_1$ and $g:B_2 \rightarrow A_2$ and $B_1$ and $B_2$ are disjoint we denote $f \cup g$ for the unique vector with domain $B_1 \cup B_2$ that agrees with both *f* and *g* on all values. We use the shorthand $b \mapsto a$ for the vector *f* with co-domain $\{b\}$ with $f(b)=a$.

The standard dynamic programming algorithm that solves *k*-choosability in $2^{q^{w}}n^{O(1)}$ time defines for each $i=1,\ldots,n$ and list assignment $$l: L_i \rightarrow ( {\begin{array}{l}[q]\\ k\end{array}})$$ the table entry $T_i[l]$ to be the set

1 $$\begin{aligned} & \Bigl \{ \{ f_{|L_i}: f \text { is a list coloring of } \,\,(G_i,l') \} \Bigm \vert l':V(G_i) \rightarrow \left( {\begin{array}{c}[q]\\ k\end{array}}\right) \nonumber \\ & \quad \text { s.t.}\,\, \forall v \in L_i, \,\, l'(v)=l(v)\Bigr \}. \end{aligned}$$

Then we have that $T_0[\epsilon ]=\{ \epsilon \}$, where $\epsilon$ denotes the empty (zero-dimensional) vector. For $i>0$, we have that $T_i[l]$ equals to

2 $$\begin{aligned} \bigcup _{\hat{l}: L_{i-1} \setminus L_i \rightarrow \left( {\begin{array}{c}[q]\\ k\end{array}}\right) } \bigcup _{ \mathcal {A} \in T_{i-1}\left[ (l \cup \hat{l})_{|L_{i-1}}\right] } \Bigl \{ \bigcup _{c \in l(v_i)} \{ (f \cup (v_i \mapsto c))_{|L_i} \vert f \in \mathcal {A}, f^{-1}(c) \cap N(v_i)=\emptyset \} \Bigr \}. \end{aligned}$$

Intuitively, this expression first iterates over all list assignments $\hat{l}$ of $L_{i-1} \setminus L_i$ and subsequently over all sets of colorings $\mathcal {A}$ that are the result of some list assignment of $V_i$ consistent with this list assignment. For each such combination, we need to extend $\mathcal {A}$ with all possible colorings $c \in l(v_i)$ of the new vertex $v_i$ (where $v_i$ is"new"in the sense that it is the only vertex in $L_i$ but not in $L_{i-1}$).

Since $T_i[l]$ is a family of subsets of vectors in $[q]^{L_i}$ we have that $|T_i[l]| \le 2^{[q]^{L_i}}$, and the number of possibilities for *l* is at most $$({\begin{array}{l}q\\ k\end{array}}) ^w \le 2^{O(w^3)}$$. Thus number of table entries is at most $2^{O(q^w)}$. Computing table entries by following (2) in the naïve way, this results in a $2^{O(q^w)}$ time algorithm. We will now show how to improve this using the idea of representation

### Definition 2

(Definition 8 from [20], paraphrased) Fix a bipartite graph *H* with parts *L* and *R*. A family $\mathcal {F}' \subseteq [q]^{L}$*H-represents* another family $\mathcal {F} \subseteq [q]^{L}$ if $\mathcal {F}' \subseteq \mathcal {F}$ and for each coloring $b \in [q]^{R}$ the following holds: there exists $a \in \mathcal {F}$ such that $a \cup b$ is a proper coloring of *H* if and only if there exists an $a' \in \mathcal {F}'$ such that $a' \cup b$ is a proper coloring of *H*.

### Theorem 12

(Lemma 10 from [20], paraphrased) For every $\mathcal {F} \subseteq [q]^{L}$, there exists an $\mathcal {F}' \subseteq \mathcal {F}$ that *H*-represents $\mathcal {F}$ such that $|\mathcal {F'}| \le 2^{|E(H)|}$. Moreover, given $|\mathcal {F}|$, such an $\mathcal {F}'$ can be computed in time $|\mathcal {F}|2^{O(w)}$, where *w* is an upper bound on the number of edges of *H*.

We refer to a subset of $2^{[q]^{L_i}}$ as a *table*. The intuition for this name is that such objects are exactly the kind of families stored in the naïve dynamic programming algorithm of $T_i$ as defined in (1).

### Definition 3

(Representation of tables) Fix a bipartite graph *H* with parts *L* and *R*. A table $T' \subseteq 2^{[q]^{L}}$*H-represents* another table $T \subseteq 2^{[q]^{L}}$ if

1. for each $\mathcal {A} \in T$ there is an $\mathcal {A}' \in T'$ that *H*-represents $\mathcal {A}$,
2. for each $\mathcal {A'} \in T'$ there is an $\mathcal {A} \in T$ such that $\mathcal {A'}$*H*-represents $\mathcal {A}$.

If *H* is clear from the context, we simply say that $T'$*representsT*. It is easy to see (and recorded as Observation 9 in [20]) that the *H*-representation relation of families of colorings is *transitive*: If $\mathcal {A}''$ represents $\mathcal {A}'$ and $\mathcal {A}'$ represents $\mathcal {A}$, then $\mathcal {A}''$ also represents $\mathcal {A}$. Using this, it is also easy to see that the *H*-representation relation of *tables* is transitive: If $T''$ represents $T'$ and $T'$ represents *T*, then $T''$ also represents *T*.

### Theorem 13

Given *T*, we can compute a table $T'$ in time $\sum _{\mathcal {A} \in T}|\mathcal {A}|2^{O(w)}$ such that $$|T'| \le \sum _{i=0}^{2^w} ({\begin{array}{l}q^w\\ i\end{array}}) \le 2^w({\begin{array}{l}q^w\\ 2^w\end{array}})$$ and $T'$*H*-represents *T*, where *w* is an upper bound on the number of edges of *H*.

### Proof

We can simply apply Theorem 12 on each member of *T*, and remove copies. $\square$

### Lemma 3

Let $$0 < i \le n$$. Suppose that $$T'_{i-1}[l]$$$$H_{i-1}$$-represents $$T_{i-1}[l]$$ for each *l*. We define $$T'_i[l]$$ as

3 $$\begin{aligned} \bigcup _{\hat{l}: L_{i-1} \setminus L_i \rightarrow ({\begin{array}{l}[q]\\ k\end{array}}) } \bigcup _{ \mathcal {A} \in T'_{i-1}[(l \cup \hat{l})_{|L_{i-1}}] } \Bigl \{ \bigcup _{c \in l(v_i)} \{ (f \cup (v_i \mapsto c))_{|L_i} \vert f \in \mathcal {A}, f^{-1}(c) \cap N(v_i)=\emptyset \} \Bigr \}. \end{aligned}$$

Then $$T'_i[l]$$$$H_i$$-represents $$T_i[l]$$ for each *l*.

### Proof

We prove the statement by induction on *i*. For $i=0$, we define $T_0[\epsilon ]=T_0'[\epsilon ]=\{ \epsilon \}$, and therefore the base case trivially holds.

Suppose that $\mathcal {A} \in T_i[l]$. Thus by the definition in (1) we have that

$$\begin{aligned} \mathcal {A} = \{ f_{|L_i}: f \text { is a list coloring of} \,\, (G_i,l') \}, \end{aligned}$$

for some $$l': V(G_i)\rightarrow ({\begin{array}{l}[q]\\ k\end{array}})$$ that agrees with *l* on all vertices in $L_i$. Consider the corresponding family $\mathcal {A}_0 \in T_{i-1}[l'_{|L_{i-1}}]$:

$$\begin{aligned} \mathcal {A}_0 = \{ f_{|L_{i-1}}: f \text { is a list coloring of} \,\, (G_{i-1},l'_{|V_{i-1}}) \}. \end{aligned}$$

Since $T'_{i-1}[l'_{|L_{i-1}}]$ by assumption $H_{i-1}$-represents $T_{i-1}[l'_{|L_{i-1}}]$, there is some $l^*$ that agrees with $l'$ on all vertices in $L_{i-1}$ such that $T'_{i-1}[l'_{|L_{i-1}}]$ contains a set

$$\begin{aligned} \mathcal {A}^*_0 = \{ f_{|L_{i-1}}: f \text { is a list coloring of} \,\, (G_{i-1},l^*) \}. \end{aligned}$$

that $H_{i-1}$-represents $\mathcal {A}_0$. We claim that the set

$$\begin{aligned} \mathcal {A}^* = \bigcup _{c \in l(v_i)} \{ (f \cup (v_i \mapsto c))_{|L_i} \vert f \in \mathcal {A}^*_0, f^{-1}(c) \cap N(v_i)=\emptyset \}, \end{aligned}$$

which is in $T'_i[l]$ by (3), $H_i$-represents $\mathcal {A}$.

First note that $\mathcal {A}^*_0 \subseteq \mathcal {A}_0$, and since $\mathcal {A}^*$ is obtained from $\mathcal {A}^*_0$ by assigning all possible colors not in $\{ l(u): u \in N(v_i)\}$ to $v_i$ it also assigns to $l(v_i)$ at some point. Thus $\mathcal {A}^* \subseteq \mathcal {A}$. Now, suppose there exists an $a \in \mathcal {A}$ and a $b \in [q]^{R_i}$ such that $a\cup b$ is a proper coloring of $H_i$. That means there is an *f* that is a list coloring of $(G,l')$ such that $f_{|L_i}=a$ and $l'$ agrees with *l* on all vertices in $L_i$. Then $f_{|L_{i-1}} \cup (f \cup b)_{|R_{i-1}}$ forms a proper coloring of the bipartite graph $H_{i-1}$. Since $f_{|L_{i-1}} \in \mathcal {A}_0$ and $\mathcal {A}^*_0$ is $H_{i-1}$-representing $\mathcal {A}_0$, there also is a $g \in \mathcal {A}^*_0$ that forms a proper coloring of $H_{i-1}$ together with $(b \cup f)_{|R_{i-1}}$. Now $g \cup (v_i \mapsto l(v_i)) \in \mathcal {A}^*$ since $\mathcal {A}^*$ is constructed in (3) by mapping *v* to each allowed color, and $(g \cup (v_i \mapsto l(v_i)))_{|L_i}$ forms a proper coloring with *b* of $H_i$. Thus $\mathcal {A}^*$ indeed represents $\mathcal {A}$.

It remains to prove item 2 of Definition 3. Suppose $\mathcal {A}' \in T'_i[l]$. Then

$$\begin{aligned} \mathcal {A}'= \bigcup _{c \in l(v_i)} \{ (f \cup (v_i \mapsto c))_{|L_i} \vert f \in \mathcal {A}'_0, f^{-1}(c) \cap N(v_i)=\emptyset \}, \end{aligned}$$

for some $$\hat{l}$$ and $$\mathcal {A}'_0 \in T'_{i-1}[(l \cup \hat{l})_{|L_i}]$$. We know that there exists an $$\mathcal {A}_0 \in T_{i-1}[(l \cup \hat{l})_{|L_i}]$$ with the property that $$\mathcal {A}'_0$$$$H_{i-1}$$-represents $$\mathcal {A}_0$$. Then, similarly as before, we have that $$\mathcal {A}'$$$$H_i$$-represents

$$\begin{aligned} \mathcal {A}= \bigcup _{c \in l(v_i)} \{ (f \cup (v_i \mapsto c))_{|L_i} \vert f \in \mathcal {A}_0, f^{-1}(c) \cap N(v_i)=\emptyset \}. \end{aligned}$$

$$\square$$

Now we are ready to combine all parts:

### Proof of Theorem 2

The algorithm is as follows: First, set $T'_0[\epsilon ]=\{\epsilon \}$. Then, for $i=1,\ldots,n$, compute $T'_i[l]$ for each $l \in [q]^{L_i}$ from the table entries $T'_{i-1}$ using Lemma 3 and before increasing *i* in each round, replace $T'_i[l]$ with a table that $H_i$-represents it and with the property that $$|T'_i[l]| \le 2^w\left( {\begin{array}{c}q^w\\ 2^w\end{array}}\right)$$ using Theorem 13.

Afterwards, we can conclude that the choice number is at least *k* if $\emptyset \notin T'_n[\epsilon ],$ since it $H_n$-represents $T_n.$ Doing this for every *k*, we solve the Choosability problem.

For the running time, the number of table entries we work with is at most $$n\cdot \left( {\begin{array}{c}q\\ k\end{array}}\right) ^w \le n 2^{q\cdot w} \le n 2^{O(w^3)}$$. Since we apply Theorem 13 each step before we compute new table entries, using (3) takes time at most $$2^w \left( {\begin{array}{c}q^w\\ 2^w\end{array}}\right) 2^{O(w^3)}$$, even when done in the naïve way. Using $$\left( {\begin{array}{c}n\\ k\end{array}}\right) \le n^k$$ and $q \le 4 w^2$, we get that

$$\begin{aligned} 2^w \left( {\begin{array}{c}q^w\\ 2^w\end{array}}\right) 2^{O(w^3)}n \le q^{w {2^w}}2^{O(w^3)}n \le 2^{(\log q) w 2^w}2^{O(w^3)}n \le 2^{2^{O(w)}}n. \end{aligned}$$

$$\square$$

## Other structural parametrizations

In algorithms of this section we use the following simple lemma.

### Lemma 4

Let *G* be a graph and *v* a vertex in the graph such that $deg(v)<k$. We claim that *G* is *k*-choosable if and only if $G\setminus v$ is *k*-choosable.

### Proof

It is obvious that if $G\setminus v$ is not *k*-choosable, then *G* is also not *k*-choosable.

We show that if $G\setminus v$ is *k*-choosable, then *G* is also *k*-choosable. First of all we assign colors to all vertices except *v* since $G\setminus v$ is *k*-choosable. After this we assign color to vertex *v*. It is possible to do so since the list of vertex *v* contains *k* different colors and $deg(v)\le k-1$. Hence, there is at least one color in which vertex *v* can be colored. $\square$

### Polynomial kernel parameterized by vertex cover

#### Theorem 3

$k$-Choosability admits a kernel with $O(w^k)$ vertices for a fixed integer *k*, where *w* is the size of a vertex cover of the input graph *G*.

#### Proof

If a vertex cover is not provided with the input, we compute a 2-approximation of a vertex cover in polynomial time and use it as a substitute for the minimum-sized vertex cover.

We need the following simple reduction rule.

**Reduction Rule 1.***If*$v\in V(G)$*and*$deg(v)<k$, *then replace graphGby graph*$G\setminus v$.

The correctness of the above rule follows from Lemma 4. It is easy to see that exhaustive application of the rule requires at most polynomial time. We note that the size of the vertex cover can only decrease after application of Reduction Rule 1 and vertex cover of the final graph equals to $C'$ without deleted vertices.

After exhaustive application of Reduction Rule 1 we partition vertices of the graph into vertex cover $C\subseteq C'$ and independent set *I*. We claim that if $$|I|\ge \left( {\begin{array}{c}w\\ k \end{array}}\right) \cdot k^k$$, then *G* is a No-instance, which immediately leads to $O(w^k)$ polynomial kernel since $|C|\le w$ and *k* is a fixed integer. In order to prove the claim we note that $deg(u)\ge k$ for all $u \in I$. We prove that if $$|I|\ge ( {\begin{array}{l}w\\ k \end{array}}) \cdot k^k$$, then *G* contains $K_{k,k^k}$ as an induced subgraph which is not *k*-choosable by Lemma 1. In order to do this, for each subset $S\subseteq C$ of size *k* we assign a subset $N_S = \{u| u\in I, S\subseteq N(u) \}$. If for some $S'$ of size *k* we have that $N_{S'}\ge k^k$, then we have found an induced subgraph $K_{k,k^k}$ (take $S'$ as one part and $k^k$ arbitrary vertices from $N_{S'}$ as a second part). Since $$\sum _{S\subseteq C}|N_S| \ge|I|\ge ( {\begin{array}{l}w\\ k \end{array}}) \cdot k^k$$ we conclude that such $S'$ exists. Hence we obtained the desired kernel with $O(w^k)$ vertices. $\square$

We note that by trivial AND-composition (see [13]) to itself we get the following result.

#### Note 1 

If $NP \not \subseteq coNP/poly$, then $k$-Choosability does not admit a polynomial kernel under parametrizations by treewidth, pathwidth, cutwidth, treedepth and all parameters *p* that satisfy $p(H_1\sqcup H_2 \sqcup \dots \sqcup H_{\ell })\le max_{i=1}^{\ell } p(H_i)+const$ where $\sqcup$ denotes disjoint union.

### Dual parameterization

#### Theorem 4

$k$-Choosability under the dual parametrization by $n-k$ can be compressed into an instance of $k$-Choosability or into an instance of Dual-Coloring. Moreover, the number of vertices in the compressed instance is at most $3(n-k)$.

#### Proof

We consider two cases: $k\le \frac{n}{2}$ and $k>\frac{n}{2}$. In the first case we can simply return the input instance, since $n\le 2 (n-k)\le 3(n-k)$.

It remains to consider the second case when $k>\frac{n}{2}$. Before we proceed we need the following claim.

#### Claim 1

$$ch(G)\le \max \{\lfloor \frac{n}{2}\rfloor,\chi (G)\}$$.

#### Proof

Indeed, if $\chi (G)>\frac{n}{2}$ then by Theorem 8 we have $ch(G)=\chi (G)$. Otherwise, we can add edges to *G* until its chromatic number becomes $\lfloor \frac{n}{2}\rfloor$. Note that addition of edges do not decrease choosability number, and by Theorem 8 choosability of the new graph is $\lfloor \frac{n}{2}\rfloor$, so the choosability number of the initial graph was at most $\lfloor \frac{n}{2}\rfloor$. $\square$

Now it is enough to check whether $\chi (G)\le k$, since in this case $\chi (G)\le k$ implies $\max \{\lfloor \frac{n}{2}\rfloor,\chi (G)\} \ge k$. Moreover, we just proved that $ch(G)\le \max \{\lfloor \frac{n}{2}\rfloor,\chi (G)\}$. So essentially we reduce our problem to Dual-Coloring. It is known [7] that Dual-Coloring admits a linear kernel with $3(n-k)$ vertices which leads to a desired compression linear in the number of vertices.

$$\square$$

### Clique-modulator parameterization

#### Theorem 5

Choosability admits an FPT-algorithm parameterized by a size of a clique-modulator *d*. For convenience, we assume that a clique-modulator of size *d* is provided with the input.

#### Proof

We note that even though we assume that a clique-modulator *D* is provided together with the input this fact does no lead to significant restriction. Since a clique-modulator is a vertex cover in the complement graph $\bar{G}$ and 2-approximation of a vertex cover can be found in polynomial time.

Denote by $C=G\setminus D$. Since *D* is a clique-modulator we have that *C* is a clique. We consider two cases: (i) $|C|\le d$, (ii) $|C|>d$.

In the first case we have $|V(G)| =|C|+|D|\le 2d$. Therefore using algorithm from Theorem 1 we find the choosability number in $\mathcal {O}^*(2^{4d^2})$ time.

In the second case we have $\chi (G)\ge|C|$ and $2\chi (G)>V(G)$. So by Theorem 8, we know that $ch(G)=\chi (G)$. Hence, it is enough to find the chromatic number of *G*. Note that $n\ge \chi (G)\ge|C|$. So in order to determine the *ch*(*G*) we simply check for each $p \in [|C|,n]$ whether $\chi (G)=p$.

For each vertex $$v\in V(G)$$, we create a list of potential colors $$\mathcal {L}{v}=\{1,2,\dots, p\}$$. Without loss of generality we may assume that vertices from the clique *C* are colored in colors $$\{1,2, \dots,|C|\}$$. After that for each vertex $$u \in D$$ we delete color *i* from the list $${{\,\mathrm{\mathcal {L}}\,}}(u)$$ if there is a vertex $$v \in C\cap N(u)$$ with color *i*. If after these modifications a vertex *w* has a list of length larger than *d*, then we delete vertex *w*. Namely, such a vertex can be easily colored after we color other vertices. We also remove all vertices from the clique *C*, as a result we get a graph $$G'$$ with list of length at most *d* for each vertex. It is easy to see that the graph $$G'$$ admits a list coloring if and only if the chromatic number of *G* was at most *p*. List Coloring can be solved in $$\mathcal {O}^*(2^d)$$ by subset convolution method [4]. So, we obtain that *ch*(*G*) can be found in $$\mathcal {O}^*(2^{4d^2}).$$$$\square$$

### Split graphs

#### Lemma 5

Choosability on split graphs can be solved in polynomial time.

#### Proof

First of all we find a representation of the split graph *G* as union of a clique *C* and an independent set *I*. Additionally, we require that *C* is the largest clique in the graph. We can find the representation in polynomial time since split graphs are chordal graphs and in chordal graphs we can find the largest clique in polynomial time [10]. In this representation degrees of all vertices from the set *I* are at most $|C|-1$. We eliminate all vertices from the set *I*. By Lemma 4 the choosability number will not change, since $ch(G)\ge|C|$. At this point we obtained a graph that contains only the clique *C*. Hence, $ch(G)=ch(C)=|C|$. $\square$

Since Choosability is solvable in polynomial time on split graphs, it is natural to investigate the parameterization by distance to split graphs. We denote by *d* the size of modulator to split graphs.

We show that there is no constant $\theta$ such that Choosability is solvable in $\mathcal {O}^*(\theta ^d)$ time. In order to do this we use the following result by Jaffke and Jansen.

#### Theorem 17

[19] There is no (universal) constant $\theta$, such that for all fixed $q \in O(1)$, *q*-Coloring on Independent + *kv* graphs can be solved in time $\mathcal {O}^*(\theta ^k)$, unless ETH fails.

#### Theorem 6

Unless ETH fails there is no constant $\theta$ such that Choosability is solvable in $\mathcal {O}^*(\theta ^d)$ time where *d* denotes the size of modulator to split graphs.

#### Proof

From Theorem 17 it follows that there is no constant $\theta$ such that Coloring is solvable in $\mathcal {O}^*(\theta ^k)$ on Independent + *kv* graphs. We construct a reduction from Coloring on Independent + *kv* graphs to Choosability on Split + *kv* graphs.

Let *G* be a graph from class Independent + *kv* on which we want to solve Coloring. We construct a new graph $G'$. In order to construct $G'$ we add *n* universal vertices to *G*, where $n=|V(G)|$. In this case a modulator to independent set in graph *G* becomes a modulator to split graph in $G'$. Hence, $d\le k$. Note that $\chi (G')\ge n=\frac{V(G')}{2}$. Therefore, from Theorem 8 it follows that $ch(G')=\chi (G')=\chi (G)+n$. It means that knowing the choosability number of $G'$ we can find the coloring number of *G*. So, we conclude that Choosability is not solvable in $\mathcal {O}^*(\theta ^d)$ time unless ETH is false. $\square$

### Parameterization by cliquewidth

Taking into account the tractability of the Choosability problem parameterized by treewidth, it is natural to consider parameters that might be significantly smaller than treewidth. Note that Choosability unlikely admits an FPT algorithm parameterized by twin-width. This is because Choosability is $\Pi ^p_2$-complete even on planar graphs [17], while the twin-width of planar graphs is at most 8. In the next theorem, we study the influence of cliquewidth on the complexity of Choosability.

#### Theorem 7

Choosability does not admit an FPT algorithm parameterized by cliquewidth unless $FPT=W[1]$.

#### Proof

It is known that Coloring is *W*[1]-hard parameterized by cliquewidth [12]. Hence, it is enough to provide a reduction from the Coloring problem parameterized by cliquewidth to the Choosability problem parameterized by cliquewidth. In order to transform an instance of Coloring to an instance of Choosability, for a given graph *G* with *n* vertices, we add *n* universal vertices. Let us call the new graph $G'$. We have that $\chi (G)+n=\chi (G')$. Since graph $G'$ has a clique of size *n*, we have that $\chi (G')\ge \frac{|V(G')|}{2}$. Therefore, from Theorem 8, it follows that $\chi (G')=ch(G').$ So, knowing the choosability number of $G'$, we can easily compute $\chi (G)=ch(G')-n$. Note that $cw(G')\le cw(G)+2$. Indeed, having an expression with *k* labels that construct graph *G*, we can construct $G'$ using at most $k+2$ labels. First of all, we construct a clique of size *n* using two labels $k+1$ and $k+2$. After that, we construct graph *G* using labels from the set $\{1,2, \dots, k\}$. Finally, we join with edges all pairs of vertices *u*, *v* such that *u* has a label from the set $\{1, 2,\dots, k\}$ and *v* has a label $k+1$ or $k+2$. As during the reduction, the parameter increased only by 2 and the whole transformation took polynomial time, we conclude that Choosability does not admit an FPT algorithm parameterized by cliquewidth unless $FPT=W[1]$. $\square$

## Conclusion

At this paper we constructed a $\mathcal {O}^*(2^{n^2})$-time algorithm for Choosability. We think that existence of algorithm with running time $\mathcal {O}^*(2^{n^{2-\epsilon }})$ for some $\epsilon>0$ for the Choosability problem is an interesting open question.

## Footnotes

1 https://cstheory.stackexchange.com/questions/2661/how-many-distinct-colors-are-needed-to-lower-bound-the-choosability-of-a-graph
